# A Shift in the Plasma 7-Oxysterol Redox Axis in Colorectal Cancer

**DOI:** 10.3390/metabo16100716

**Published:** 2026-09-26

**Authors:** Muhammad Kamran, Irundika H. K. Dias, Akinfemi Akingboye, Theodoros Kantidakis

**Affiliations:** 1 Aston Medical School, School of Medicine, Pharmacy and Biosciences, Aston University, Birmingham B4 7ET, UKdiashki1@aston.ac.uk (I.H.K.D.); a.akingboye@nhs.net (A.A.); 2 The Dudley Group NHS Foundation Trust, Russells Hall Hospital, Dudley DY1 2HQ, UK

**Keywords:** oxysterols, colorectal cancer, HSD11B2, HSD11B1, 7-ketocholesterol, 7β-hydroxycholesterol, GPR183, lipid metabolism, metabolomics, redox metabolism

## Abstract

**Background/Objectives**: Colorectal cancer (CRC) involves metabolic reprogramming, including altered cholesterol handling and redox homeostasis. Oxysterols link lipid metabolism with redox and immune regulation, but whether defined oxysterol redox systems are perturbed in the circulation is unclear. One such system is the 7-ketocholesterol (7-KC)/7β-hydroxycholesterol (7β-OHC) couple, interconverted by 11β-hydroxysteroid dehydrogenases (HSD11B1 and HSD11B2). **Methods**: We combined targeted LC-MS/MS profiling of plasma (CRC, *n* = 6; controls, *n* = 20) and paired tumour-normal tissues (10 pairs) with TCGA COAD/READ transcriptomes (normal, *n* = 51; tumour, *n* = 624). Oxysterols were normalised to cholesterol. Rank-based statistics, effect sizes, leave-one-out resampling and Benjamini–Hochberg correction were applied. **Results**: CRC plasma showed elevated hydroxylated 7-oxysterols (7β-OHC, ~2-fold, q = 0.002; 7α,25-dihydroxycholesterol, ~2.1-fold, q = 0.001). The side-chain oxysterols 25-OHC and 27-OHC were unchanged. Median 7-KC was markedly lower in CRC, but the individual comparison was not statistically significant (q = 0.263) in the context of substantial heterogeneity among healthy controls. The 7-KC/7β-OHC ratio was reduced ~23-fold (log_2_FC = −4.5, 95% CI −5.0 to −1.6; q = 0.001). This reduction reflects the individually non-significant decrease in 7-KC together with the independently significant increase in 7β-OHC. The shift was consistent across all CRC samples and robust to resampling. In contrast, paired tumour tissues were heterogeneous with no consistent change. TCGA tumours showed downregulation of oxysterol pathway genes, most prominently *HSD11B2* (~5-fold), which oxidises 7β-OHC to 7-KC. **Conclusions**: CRC is associated with a selective shift in the plasma 7-oxysterol redox axis that is directionally consistent with *HSD11B2* suppression in CRC transcriptomes, but not mirrored by steady-state tumour oxysterol levels. The 7-KC/7β-OHC ratio provides a measure of the balance between these biochemically linked circulating oxysterols. Given the small plasma cohort, these findings warrant validation in larger, prospectively matched cohorts before their broader or clinical significance can be established.

## 1. Introduction

Colorectal cancer (CRC) is characterised by extensive metabolic reprogramming, including alterations in lipid metabolism and redox homeostasis [1,2,3]. Among these changes, disrupted cholesterol metabolism has attracted increasing attention. CRC cells exhibit altered cholesterol biosynthesis, enhanced lipoprotein uptake and remodelled cholesterol efflux pathways, all of which contribute to tumour growth and progression [3,4,5]. However, it remains unclear how these tumour-associated changes are reflected in the systemic circulation and whether circulating lipid metabolites can report on underlying enzymatic processes.

Oxysterols, oxygenated derivatives of cholesterol generated through both enzymatic and non-enzymatic pathways, are bioactive signalling molecules that link cholesterol homeostasis, redox balance, inflammation and immunity [6,7,8,9]. Beyond their individual activities, certain oxysterols participate in defined biochemical systems shaped by enzymatic interconversion and redox state. Among these, 7-ketocholesterol (7-KC) and 7β-hydroxycholesterol (7β-OHC) form a biochemically defined redox couple interconverted by 11β-hydroxysteroid dehydrogenase enzymes: HSD11B1 catalyses the NADPH-dependent reduction of 7-KC to 7β-OHC, whereas HSD11B2 catalyses the reverse NAD^+^-dependent oxidation [10,11,12]. Because 7-KC arises from oxidative cholesterol modification yet can be enzymatically reduced, the 7-KC/7β-OHC ratio may be influenced by both oxidative formation and enzymatic interconversion, rather than the abundance of either metabolite in isolation [5,9,13]. This raises the possibility that the ratio may provide a measure of the balance between these two biochemically linked oxysterols.

In CRC, expression of oxysterol-metabolising enzymes is altered at the tumour level. HSD11B2 has attracted particular interest because of its dual role in glucocorticoid inactivation and 7-oxysterol oxidation, with evidence of reduced expression in established carcinomas [14,15,16]. At the metabolite level, studies have identified selective intra-tumoural accumulation of specific oxysterols, such as 27-hydroxycholesterol in advanced disease [17], and prospective analyses have linked circulating oxysterols to adenoma risk [18]. However, these studies have largely focused on individual metabolites or tumour-local processes. Whether tumour-associated changes in oxysterol metabolism are accompanied by a measurable shift in defined oxysterol redox systems in the circulation in established CRC remains unclear. In addition, 7α,25-dihydroxycholesterol (7α,25-diOHC), a potent ligand for the immune receptor EBI2/GPR183 [19,20,21], has not been examined in the circulation in CRC. Because circulating oxysterol concentrations can also be shaped by systemic influences, including dietary exposure to cholesterol oxidation products, lipid-lowering therapy and hepatic sterol metabolism [11,13,22,23,24], plasma and tumour tissue provide distinct but complementary views of oxysterol biology.

Here, building on prior LC-MS/MS-based oxysterol profiling approaches [17,25,26], we examined the 7-oxysterol redox axis in CRC by combining targeted LC-MS/MS profiling of plasma and paired tumour-normal tissue oxysterols, including 7α,25-diOHC, with analysis of tumour transcriptomes from TCGA COAD/READ. We asked whether CRC is associated with a measurable shift in this defined redox system in the circulation and whether such a shift is reflected in tumour oxysterol composition or instead dissociated from tumour-local metabolite changes.

## 2. Materials and Methods

### 2.1. Study Cohort and Samples

Paired CRC tumour-normal tissue samples (*n* = 10 pairs) and matched patient plasma were obtained via the Arden Tissue Bank (Coventry, UK). Matched plasma was available for 7 of the 10 patients. One of these (J58110), with clinically diagnosed liver cirrhosis, was excluded from plasma analyses because of the expected confounding effects of liver disease on sterol metabolism, but was retained for tissue analyses, leaving *n* = 6 for plasma. Healthy plasma controls (*n* = 20) were obtained from adult volunteer donors without known malignancy (median age 73 years, range 69–79; 9 male, 11 female). The analysed plasma CRC cohort (*n* = 6; 4 male, 2 female) spanned ages 36–87 years (median 66) and included both early-stage (I-II, *n* = 3) and late-stage (III-IV, *n* = 3) disease. Age (Mann–Whitney U, *p* = 0.298) and sex distribution (Fisher’s exact, *p* = 0.645) did not differ significantly between CRC cases and controls. Given the small CRC cohort, these non-significant comparisons should not be interpreted as establishing demographic equivalence. Available individual demographic and clinical characteristics of the study cohorts are provided in Table S1. Clinical and metabolic information beyond age and sex was not available for the healthy controls. Healthy-control plasma was separated within approximately 15 min of blood collection using the previously described protocol [24], whereas CRC plasma was supplied by the Arden Tissue Bank as previously separated plasma; full upstream collection details for the CRC samples were not available. Plasma samples were aliquoted before freezing, stored at −80 °C for at least six months, and underwent a single freeze–thaw cycle before analysis.

### 2.2. LC-MS/MS Analysis

Oxysterols (25-OHC, 27-OHC, 7β-OHC, 7-KC, 7α,25-diOHC) were quantified by targeted LC-MS/MS as previously described [17,25,26]. Briefly, plasma aliquots (70 µL) were spiked with deuterated internal standards, precipitated with methanol containing butylated hydroxytoluene (50 µg/mL), centrifuged (14,000× *g*, 10 min), diluted to 10% methanol and purified by solid-phase extraction. Tissue samples (~50 mg) were homogenised in methanol with internal standards, centrifuged and processed in parallel. Oxysterols were eluted with butyl acetate, dried under vacuum and reconstituted in methanol:water (40:60, *v*/*v*); internal standard recovery ranged from 72% to 82%. LC-MS/MS used an UltiMate 3000 HPLC (Thermo Fisher Scientific, Hemel Hempstead, UK) coupled to a QTRAP 5500 mass spectrometer (SCIEX, Warrington, UK) with C18 separation (NUCLEOSIL^®^ C18, 100 mm, 5 µm pore size; Macherey-Nagel, Düren, Germany) using methanol:water and isopropanol:methanol mobile phases, 0.1% formic acid at 200 µL/min flow rate at 45 °C. The analytical method was previously validated [24]. Calibration was linear over 0.01–10 ng per injection (R^2^ ≥ 0.99), with intra- and inter-day precision within 15%. For 7-KC, intra- and inter-day coefficients of variation ranged from 1.2% to 10.4%, with errors of approximately 1–3%, while corresponding coefficients of variation for 7β-OHC ranged from 0.8% to 10.3% [24]. The published limits of detection (LOD) and lower limits of quantification (LLOQs) were 24/122 pg/mL for 25-OHC, 44/115 pg/mL for 27-OHC, 5/18 pg/mL for 7β-OHC and 12/39 pg/mL for 7-KC. For 7α,25-diOHC, the corresponding LOD/LLOQ values were 16/45 pg/mL, determined in the same laboratory using the same analytical platform and workflow. No plasma measurement fell below its corresponding LOD, and all 7-KC and 7β-OHC measurements in both groups were above their respective LLOQs. A single 25-OHC measurement in one healthy control fell slightly below the LLOQ but remained well above the LOD and was retained at its measured value; no values were substituted or censored. CRC and healthy-control plasma were processed and analysed as separate sample sets using the same LC-MS/MS workflow. Within each set, sample order was randomised and a pooled QC sample was analysed after every five samples. Oxysterol concentrations were normalised to cholesterol. The 7-KC/7β-OHC ratio was calculated as the cholesterol-normalised concentration of 7-KC divided by that of 7β-OHC.

### 2.3. TCGA Transcriptomic Analysis

TCGA COAD/READ RNA-seq data were obtained via UCSC Xena [27]. Nine genes with established biochemical roles in 7-oxysterol interconversion (*HSD11B1*, *HSD11B2*), synthesis (*CH25H*, *CYP27A1*, *CYP7B1*), signalling (*NR1H3*, *NR1H2*) and efflux/conjugation (*ABCA1*, *SULT2B1*) were analysed. Expression values were log_2_(FPKM-UQ + 1) transformed. Where patients had multiple tumour samples, expression values were averaged per patient before unpaired comparisons. Log_2_ fold changes were calculated as the difference between mean log_2_ (FPKM-UQ + 1) values in tumour and normal groups. Median-based fold changes were additionally computed as a robustness check (Table S2). Differential expression was assessed using Mann–Whitney U tests for unpaired comparisons and Wilcoxon signed-rank tests for paired comparisons, with Benjamini–Hochberg false discovery rate correction [28]. To assess consistency across clinical subgroups, analyses were repeated with tumours stratified by microsatellite instability status (MSS vs. MSI-H, based on TCGA molecular subtype annotations) and by tumour stage (early: stages I–II; late: stages III–IV), in each case compared against the full normal sample set as a common baseline, with Benjamini–Hochberg correction applied separately across the nine-gene panel within each stratum. To test whether expression differed directly between clinical subgroups, patient-level tumour expression was additionally compared between MSI-H and MSS tumours and between late-stage (III–IV) and early-stage (I–II) tumours using two-sided Mann–Whitney U tests. Where multiple tumour aliquots were available for an individual patient, expression values were averaged before analysis. Cliff’s δ was calculated as an effect-size measure. For each direct clinical comparison, *p* values were Benjamini–Hochberg adjusted across the nine-gene oxysterol panel.

### 2.4. CPTAC Proteomic Validation

HSD11B2 protein abundance was analysed in the CPTAC colon adenocarcinoma (COAD) cohort [29] using processed data accessed through the cptac Python package (version 1.5.14) [30]. Proteomic and transcriptomic datasets were used. Proteomic abundance was represented as log_2_ reference-normalised intensity and transcript abundance as log_2_(UQ-normalised RSEM + 1). Normal and tumour proteomic samples were matched by patient identifier; where repeated aliquots were present, values were averaged to obtain one value per patient. HSD11B2 protein abundance was compared between matched tumour and normal tissue using a two-sided Wilcoxon signed-rank test. The median paired tumour-normal difference and its 95% confidence interval were estimated using 10,000 bootstrap resamples. HSD11B2 mRNA-protein association within tumours was assessed using Spearman rank correlation. *p* values for the two CPTAC validation analyses were adjusted together using the Benjamini–Hochberg procedure. Normal transcriptomic data were not available in the processed CPTAC COAD dataset used here. Secondary subgroup analyses examined HSD11B2 according to tumour stage and microsatellite instability status. HSD11B2 tumour transcript and protein abundance were compared directly between early-stage (I–II) and late-stage (III–IV) disease using two-sided Mann–Whitney U tests. For the MSI analysis, the patient-level paired tumour-normal difference in HSD11B2 protein abundance was calculated and compared between MSI-H and MSS cases using a two-sided Mann–Whitney U test. *p* values for these three secondary comparisons were adjusted together using the Benjamini–Hochberg procedure.

### 2.5. Statistical Analysis

Given the small cohort sizes and limited ability to assess normality reliably, rank-based non-parametric tests were used. Plasma comparisons were performed using two-sided Mann–Whitney U tests, whereas tissue comparisons used two-sided Wilcoxon signed-rank tests on paired samples. Effect sizes were quantified using Cliff’s δ [31] for unpaired comparisons and matched-pairs rank-biserial correlation for paired comparisons; both range from −1 to +1, where values near the extremes indicate near-complete separation in one direction. For unpaired comparisons, Cliff’s δ is numerically equivalent to the rank-biserial correlation, and effect sizes are therefore reported interchangeably where appropriate. Plasma log_2_ fold changes were calculated as log_2_(median CRC/median control). For paired tissue samples, fold changes were calculated as log_2_[(tumour + p)/(normal + p)], where p = 9.1 × 10^−4^, corresponding to half the lowest non-zero concentration measured across the tissue analytes; p was added to all tissue concentrations before any ratio was formed. Bootstrap 95% confidence intervals (CI; 10,000 resamples; percentile method) were computed for plasma fold changes and for effect sizes in both plasma and tissue analyses. Leave-one-out sensitivity analysis sequentially excluded each healthy control and each CRC case, with the CRC-control difference in the 7-KC/7β-OHC ratio re-tested after each exclusion. As an additional sensitivity analysis, J58110 was re-included and the six plasma comparisons were repeated with Benjamini–Hochberg false discovery rate correction. Associations were assessed using Spearman’s rank correlation. Multiple testing was controlled using the Benjamini–Hochberg false discovery rate, applied separately across the six plasma and six tissue comparisons. Principal component analysis was performed using only the five directly measured, cholesterol-normalised plasma oxysterols (25-OHC, 27-OHC, 7β-OHC, 7-KC and 7α,25-diOHC), after mean-centring and scaling each variable to unit variance. The derived 7-KC/7β-OHC ratio was not included in the PCA. PCA loadings were calculated for each oxysterol to assess the contribution of individual metabolites to the principal components. The ratio was included only in the separate Spearman correlation analysis. All analyses were performed in Python (v3.10.19) using NumPy, pandas, SciPy and scikit-learn, with figures generated in matplotlib and seaborn. During the preparation of this work, the authors used Claude (Anthropic) to assist with Python analysis script debugging and development. All code was executed and verified locally, and all outputs were reviewed and edited by the authors, who take full responsibility for the content of this article.

## 3. Results

### 3.1. A Shift in the Circulating 7-Oxysterol Redox Axis in CRC Plasma

We compared plasma oxysterol profiles between healthy controls (*n* = 20) and CRC patients (*n* = 6). Cholesterol showed a non-significant trend towards lower levels in CRC (*p* = 0.072; Figure S1) and was used to normalise oxysterol abundance to the available cholesterol pool. This allowed subsequent comparisons to focus on oxysterol redistribution relative to circulating cholesterol rather than on absolute cholesterol differences.

CRC plasma exhibited a pronounced redistribution within the 7-oxysterol pool. Hydroxylated 7-oxysterols were consistently elevated; 7β-hydroxycholesterol (7β-OHC) was increased approximately 2.0-fold (95% CI 1.6 to 3.0; q = 0.002) and 7α,25-dihydroxycholesterol (7α,25-diOHC) approximately 2.1-fold (95% CI 1.5 to 2.9; q = 0.001), both with large effect sizes (Figure 1C,E and Figure S2). In contrast, the side-chain oxysterols 25-OHC and 27-OHC, which arise through distinct enzymatic pathways, were unchanged (Figure 1A,B). Because a lower cholesterol denominator would tend to increase all cholesterol-normalised oxysterol values, the selective elevation of 7β-OHC and 7α,25-diOHC, with unchanged 25-OHC and 27-OHC, cannot be explained by the modest trend towards lower plasma cholesterol in CRC. A sample-level heatmap confirmed that these shifts were consistent across all CRC samples and were not driven by outliers (Figure S3).

The 7-KC showed a different pattern (Figure 1D). All CRC patients had uniformly low circulating 7-KC, but the CRC-control comparison did not reach statistical significance (q = 0.263), with substantial heterogeneity among healthy controls whose values spanned a wide range (Figure 1D). The clearest circulating signal emerged when these metabolites were integrated into the 7-KC/7β-OHC ratio (Figure 1F and Figure S4B), which describes the relative balance between oxo- and hydroxy-7-oxysterol species. This ratio was reduced approximately 23-fold in CRC (log_2_FC = −4.5, 95% CI −5.0 to −1.6; q = 0.001; Figure 1F,G and Figure S2), representing the largest effect observed across all measured variables, with uniformly negative fold changes across CRC patients. Because the ratio reflects the relative balance of 7-KC and 7β-OHC, rather than their cholesterol-normalised abundance, the modest, non-significant trend toward lower cholesterol in CRC plasma does not explain this result. The magnitude and consistency of the shift are consistent with a change in the relative balance within the 7-oxysterol redox couple in this cohort.

A forest plot of log_2_ fold-changes summarised the plasma phenotype, showing three FDR-significant alterations: increased 7β-OHC, increased 7α,25-diOHC and a marked reduction in the 7-KC/7β-OHC ratio (Figure 1G); for all three, the 95% confidence interval excluded zero. By contrast, the estimated log_2_ fold changes for the side-chain oxysterols 25-OHC and 27-OHC were close to zero, with confidence intervals spanning zero (Figure 1G), supporting the selectivity of the phenotype within the 7-oxygenated pool. Although 7-KC did not reach significance individually, CRC values were uniformly low and the median was markedly lower than in controls (Figure 1D), with the wide dispersion among control values limiting the precision of the group comparison.

Across individual samples, all CRC patients exhibited increased 7β-OHC and 7α,25-diOHC together with a reduced 7-KC/7β-OHC ratio, with limited overlap with the control distribution (Figure 1C,E,F). Principal component analysis showed that PC1 and PC2 explained 44.4% and 32.2% of the variance, respectively (76.6% cumulatively), with PC1 largely separating CRC samples from controls (Figure S5A). PC1/PC2 loadings showed contributions from several oxysterols, indicating that the multivariate structure was not dominated by a single metabolite (Figure S5B). The 7-KC/7β-OHC ratio correlated positively with 7-KC (ρ = 0.74) and inversely with 7β-OHC (ρ = −0.55; Figure S5C), indicating that both arms of the couple contributed to the shift. Because the fall in 7-KC was proportionally larger (median roughly 13-fold lower in CRC) than the rise in 7β-OHC (~2-fold higher), the reduction in the ratio reflected a greater contribution from 7-KC depletion than from 7β-OHC accumulation. A pool-level analysis supported this interpretation, as the hydroxylated 7-oxysterol pool (7β-OHC + 7α,25-diOHC) was significantly elevated in CRC plasma, whereas the oxo pool (7-KC) alone was unchanged, resulting in a modest but significant increase in total measured 7-oxysterols (Figure S6A).

Given the variability within the control group, we performed leave-one-out (LOO) resampling to assess robustness. Sequential exclusion of each healthy donor, followed by re-testing of the CRC-control difference, showed that the 7-KC/7β-OHC ratio separation remained highly significant in every iteration (*p* range: 4.5 × 10^−5^ to 2.2 × 10^−4^; Figure S4A). Exclusion of individual CRC patients likewise preserved significance (*p* range: 2.6 × 10^−4^ to 7.2 × 10^−4^; Figure S4A). When healthy controls were stratified at their median 7-KC/7β-OHC ratio to visualise within-group heterogeneity, CRC values lay at or below the lower control subgroup (Figure S4B). Re-inclusion of J58110 had minimal effect on the result: although this patient had a higher 7-KC/7β-OHC ratio than the median of the six CRC cases included in the primary analysis (0.0447 vs. 0.0201), the CRC median remained 0.0204 compared with 0.4588 in healthy controls, corresponding to an approximately 22.5-fold reduction (log_2_FC = −4.49; q = 0.0009). The observed separation therefore remained robust to single-sample exclusion and was not attributable to any individual sample.

Together, these findings define a coherent circulating phenotype in CRC plasma characterised by expansion of hydroxylated 7-oxysterols and a pronounced shift within the 7-KC/7β-OHC redox couple. This pattern is not explained by a uniform increase across all measured oxysterols, but instead reflects redistribution within the biochemically defined redox couple.

### 3.2. Tumour Tissue Shows Heterogeneous Oxysterol Profiles

We next asked whether tumour tissue exhibited an analogous metabolic shift. Paired tumour and adjacent normal colon (*n* = 10 pairs) were analysed using the same oxysterol panel. Unlike plasma, tissue oxysterols showed no consistent directional change (Figure 2A–F); some tumours exhibited modest increases in certain oxysterols, others showed decreases and several were largely unchanged. Wilcoxon signed-rank testing confirmed the absence of significant cohort-level shifts for any oxysterol, indicating that paired tumour tissue did not show a uniform oxysterol phenotype comparable to that observed in plasma.

A per-patient heatmap of log_2_(tumour/normal) fold changes illustrated this heterogeneity (Figure 2G). Some tumours showed broad increases across most oxysterols, others broad decreases, and the remainder mixed patterns; the tissue 7-KC/7β-OHC ratio showed the same variability. This contrasts with the relatively uniform plasma phenotype (Figure S3) and argues against a simple model in which circulating changes directly mirror steady-state tumour oxysterol abundance.

### 3.3. The Circulating 7-KC/7β-OHC Phenotype Is Dissociated from Matched Steady-State Tumour Tissue

To compare the two compartments directly, we focused on the 7-KC/7β-OHC ratio, which showed the clearest circulating signal, in the six patients with matched plasma and tissue (Figure 3A). In plasma, all six patients showed a marked reduction in the ratio relative to the healthy control median. In the same six patients, the tissue ratio expressed relative to each patient’s adjacent normal tissue was variable and showed no consistent direction across tumours (Figure 3A).

We therefore compared plasma and tissue by estimating effect sizes independently within each compartment (Figure 3B). For plasma (CRC vs. healthy controls), 7β-OHC, 7α,25-diOHC and the 7-KC/7β-OHC ratio showed large, directionally consistent effects whose bootstrap 95% confidence intervals excluded the null (|r| = 0.83–0.92). For tissue (tumour vs. adjacent normal), all oxysterol effect sizes were small, with confidence intervals spanning the |r| ≤ 0.47 region and including zero, indicating no consistent directional shift. Consistent with this, plasma-tissue Spearman correlations across oxysterols in the six matched patients showed no consistent positive association (Figure S6B); given the sample size, this analysis is descriptive only.

Together, these observations indicate that, within the available matched cohort, the strong, uniform plasma phenotype has no clear counterpart in steady-state tumour tissue. In Figure 3A, the tissue ratio median was modestly below zero, indicating a weak trend in the same direction as plasma; however, the tissue changes were variable across patients and substantially less consistent than the circulating phenotype. Smaller or mixed-direction tissue effects therefore cannot be excluded.

### 3.4. Transcriptional Suppression of Oxysterol Pathway Genes in TCGA COAD/READ

To examine tumour-associated alterations in oxysterol metabolism, we examined expression of nine oxysterol-related genes in TCGA COAD/READ [27,32], spanning 7-oxysterol interconversion (*HSD11B1*, *HSD11B2*), synthesis (*CH25H*, *CYP27A1*, *CYP7B1*), signalling (*NR1H3*, *NR1H2*) and efflux/conjugation (*ABCA1*, *SULT2B1*). Both unpaired (normal *n* = 51; tumour *n* = 624) and paired (*n* = 50) analyses were performed.

Tumours exhibited broad suppression across this pathway (Figure 4A; Table S2). The most prominent change was downregulation of *HSD11B2* (log_2_FC = −2.45, ~5-fold reduction; FDR q < 0.0001), the enzyme responsible for oxidation of 7β-OHC to 7-KC, consistent with previous reports of reduced *HSD11B2* expression in CRC [14,15]. This directional change is compatible with a shift favouring 7β-OHC over 7-KC. However, the TCGA cohort was independent of the metabolite cohort and therefore does not establish a direct relationship between tumour *HSD11B2* expression and the circulating phenotype.

Additional pathway components were also downregulated, although with smaller effect sizes: *CYP7B1* (mean log_2_FC = −0.23), the LXR receptors *NR1H3* (−0.91) and *NR1H2* (−0.45), and the efflux transporter *ABCA1* (−0.84). CYP7B1 participates in oxysterol metabolism, whereas NR1H3/NR1H2 and ABCA1 represent oxysterol-responsive signalling and cholesterol/oxysterol efflux pathways. These changes indicate that transcriptional suppression in CRC extends beyond *HSD11B2* to multiple components of oxysterol synthesis, signalling and export.

In contrast, *HSD11B1*, which catalyses the reverse reaction, showed no consistent alteration; a modest effect in unpaired analysis (q = 0.007) was not retained in paired comparisons (q = 0.29; Figure 4B). This indicates that *HSD11B1* expression is not robustly altered across analyses in this cohort, consistent with prior reports of heterogeneous 11β-HSD expression in colorectal cancer [14]. Together with the marked suppression of *HSD11B2*, this pattern indicates an imbalance between the two enzymes of the 7-oxysterol redox couple.

To determine whether tumour-associated HSD11B2 suppression was also evident at the protein level, we analysed the independent CPTAC colorectal cancer cohort [29]. HSD11B2 protein abundance was reduced in tumour tissue relative to matched normal tissue in 96 paired cases, with 94 of 96 tumours showing lower abundance (median tumour-normal difference, −1.41 log_2_; approximately 2.65-fold reduction; Wilcoxon signed-rank q = 4.88 × 10^−17^; Figure 4C). In tumours with matched transcriptomic and proteomic measurements, HSD11B2 mRNA and protein abundance were positively correlated (Spearman ρ = 0.486, q = 5.32 × 10^−7^; *n* = 96; Figure 4D). These data provide independent protein-level support for tumour-associated HSD11B2 suppression in CRC, but do not establish a relationship with the circulating 7-KC/7β-OHC ratio because the CPTAC and metabolite cohorts are independent.

Stratification by microsatellite instability (MSI) status showed suppression of several pathway components in both subtypes, with greater tumour-versus-normal reductions in MSI-H for several genes (Figure S7A). The largest numerical difference was observed for *HSD11B2* (log_2_FC = −3.43 in MSI-H vs. −2.23 in MSS; ~11-fold vs. ~5-fold reduction), whereas *HSD11B1* showed no consistent suppression in MSI-H (log_2_FC = +0.10), consistent with the asymmetry between the two enzymes of the 7-oxysterol redox couple being more pronounced in this subtype. Direct patient-level comparison confirmed significantly lower *HSD11B2* expression in MSI-H than MSS tumours (mean difference = −1.20 log_2_ units; q = 3.04 × 10^−12^; Figure S7B; Table S3). *CYP27A1*, which generates 27-hydroxycholesterol, likewise showed a greater tumour-versus-normal reduction in MSI-H (log_2_FC = −1.92 vs. −0.88) and was significantly lower in the direct subgroup comparison (mean difference = −1.04 log_2_ units; q = 1.26 × 10^−10^; Figure S7B; Table S3). In contrast, *HSD11B2* showed similar tumour-versus-normal suppression in early (stage I-II) and late (stage III-IV) disease (log_2_FC = −2.52 vs. −2.36) and did not differ significantly between early- and late-stage tumours in the direct comparison (mean difference = +0.17 log_2_ units; q = 0.209; Figure S7A,B; Table S3). Secondary CPTAC subgroup analyses were broadly consistent with these TCGA findings. HSD11B2 transcript and protein abundance did not differ significantly between early- and late-stage tumours (q = 0.107 for both comparisons), and HSD11B2 protein suppression was numerically greater in MSI-H than MSS tumours, although the direct subgroup comparison did not remain significant after correction for multiple testing (*p* = 0.018; q = 0.054; Figure S8).

Together, these data identify suppression of *HSD11B2* as a consistent tumour-associated feature of CRC, supported at both transcript and protein levels. However, the TCGA and CPTAC datasets and the oxysterol measurements were generated from independent cohorts. These findings should therefore be interpreted as contextual rather than causal evidence, and do not establish that tumour HSD11B2 suppression drives the circulating 7-KC/7β-OHC phenotype.

## 4. Discussion

To our knowledge, this study is the first to characterise the circulating 7-KC/7β-OHC redox couple in established colorectal cancer (CRC) and to assess circulating 7α,25-diOHC. We identify a marked shift in this axis, with elevated circulating hydroxylated 7-oxysterols (7β-OHC and 7α,25-diOHC) and a ~23-fold reduction in the 7-KC/7β-OHC ratio. The phenotype was observed across all CRC patients despite considerable variability among controls, demonstrating a consistent shift in the circulating 7-KC/7β-OHC balance within this cohort.

These findings distinguish circulating oxysterol remodelling from tumour-local oxysterol biology. We observed no consistent tumour-local shift in the 7-oxysterol redox axis; instead, plasma showed a coordinated redistribution within the 7-KC/7β-OHC couple, while paired tumour tissues were heterogeneous. This dissociation indicates that the circulating phenotype is not a passive readout of steady-state tumour oxysterol content and is consistent with integrated contributions from multiple metabolic compartments. It does not exclude a tumour contribution, as tissue steady-state levels do not necessarily reflect local synthesis, interconversion or efflux. The circulating pool may therefore integrate metabolic processes occurring across several tissues, including the liver, adipose tissue and circulating immune cells, rather than directly reflecting oxysterol abundance within the primary tumour. Systemic inflammation and metabolic stress could alter hepatic sterol handling, HSD11B-dependent interconversion, lipoprotein transport or clearance, while gut-liver signalling, potentially including microbiome-derived influences, may further influence hepatic oxysterol metabolism. Hepatic HSD11B1 may be particularly relevant because 7-KC can undergo rapid hepatic reduction to 7β-OHC through this enzyme [11]. Such changes in systemic production, interconversion or turnover could produce a plasma phenotype without requiring parallel accumulation or depletion of the same oxysterols in tumour tissue. Among oxysterols, the 7-KC/7β-OHC couple is unusual in forming a biochemically defined redox pair, with interconversion mediated by NADPH-dependent HSD11B1 and NAD^+^-dependent HSD11B2 [9,10,11]. Although 7-KC is often viewed as a marker of oxidative stress [9,13], its steady-state abundance reflects both oxidative formation and reductive clearance, and may therefore be influenced by enzymatic interconversion as well as oxidative load. The consistently low 7-KC values in CRC, together with elevated 7β-OHC, suggest that the balance within the couple favours the hydroxylated species. This pattern is compatible with impaired HSD11B2-mediated oxidation, altered cofactor availability or other systemic metabolic processes, although the circulating ratio cannot be interpreted as a direct measure of cellular redox state or HSD11B activity.

An alternative explanation for the observed plasma phenotype is inter-individual variation in dietary intake of cholesterol oxidation products or in hepatic reductive capacity, both of which are known to influence circulating 7-KC [13,22,23]. Dietary intake was not available in this cohort, and hepatic metabolic capacity was not directly assessed; contributions from these factors therefore cannot be excluded. However, several features argue against these factors being sufficient to explain the findings. CRC samples showed a consistent directional shift across all patients despite substantial heterogeneity among controls, and leave-one-out resampling confirmed that the CRC-control separation was not driven by individual samples. The dominant signal arose from the 7-KC/7β-OHC ratio rather than from absolute metabolite concentrations, indicating redistribution within a defined biochemical couple rather than a general increase in oxidised sterols. In addition, side-chain oxysterols such as 25-OHC and 27-OHC, which are likewise influenced by metabolic and dietary factors, were unchanged. Together, these observations are consistent with a selective shift within the 7-oxysterol system, although nonspecific variation in oxysterol exposure or handling cannot be excluded. Controlled dietary and metabolic studies will be required to formally assess these contributions.

The specificity of the shift within the 7-oxygenated pool also provides clues to the processes underlying it. The opposing directions of 7-KC and 7β-OHC are not readily explained by a simple increase in non-enzymatic cholesterol oxidation alone: 7β-OHC rose while 7-KC fell, markedly altering the balance between them. Consistent with this, 7α,25-diOHC is produced enzymatically through sequential CH25H and CYP7B1 activity rather than by autoxidation, yet increased alongside 7β-OHC. Together, these observations argue against non-enzymatic oxidation being the sole explanation for the pattern observed.

The TCGA analysis provides transcriptional context consistent with the circulating phenotype, rather than direct causal evidence for it. *HSD11B2*, which oxidises 7β-OHC to 7-KC, was consistently suppressed in CRC tumours, whereas *HSD11B1* was not robustly altered (Figure 4A,B). Independent CPTAC proteomic data further showed marked suppression of HSD11B2 protein in CRC tumours, supporting the tumour-associated alteration at the protein level (Figure 4C,D). This enzymatic asymmetry could, if present beyond the tumour, drive the 7-oxysterol couple towards hydroxylated species in the same direction as the circulating shift. However, the tissue metabolite data did not show the same redox shift seen in plasma. Thus, tumour *HSD11B2* suppression is best viewed as molecular evidence that this pathway is altered in CRC, rather than as proof that tumour *HSD11B2* directly determines circulating oxysterol levels. The significantly lower *HSD11B2* expression in MSI-H than MSS tumours in the direct subgroup comparison raises the possibility that altered 7-oxysterol and glucocorticoid metabolism may be particularly relevant in this immunologically distinct subtype [16,33] (Figure S7B; Table S3). In contrast, *HSD11B2* did not differ significantly between early- and late-stage tumours, despite marked tumour-versus-normal suppression in both groups, indicating that the cross-sectional data do not support a progressive stage-associated change in *HSD11B2* expression (Figure S7A,B; Table S3). The downregulation of *ABCA1* and *SULT2B1* indicates broader disruption of oxysterol export and conjugation, extending the transcriptional remodelling beyond the HSD11B enzymes.

*CYP27A1*, which catalyses 27-hydroxylation of cholesterol, was also significantly lower in MSI-H than MSS tumours in the direct subgroup comparison (Figure S7B; Table S3). At first sight this appears to conflict with previous evidence of intra-tumoural 27-OHC accumulation in stage III CRC, reported using the same LC-MS/MS platform in an MSI-unstratified cohort [17]. However, reduced expression of a synthetic enzyme would not necessarily lower steady-state 27-OHC, which reflects the net balance of synthesis and clearance. This apparent discordance therefore reinforces, rather than contradicts, the broader theme that steady-state oxysterol levels are not a simple readout of the expression of any single enzyme. In the present study, circulating 27-OHC was unchanged, distinguishing the circulating 7-KC/7β-OHC phenotype from previously reported tissue-local or adenoma-associated 27-OHC signals [18].

The elevation of 7α,25-diOHC may have additional biological implications. As a potent endogenous ligand for EBI2/GPR183, this oxysterol regulates immune cell positioning within lymphoid tissues and sites of inflammation [19,20,21]. Increased circulating levels could therefore influence immune cell trafficking in CRC, although this remains to be tested directly. Notably, *CYP7B1*, which contributes to 7α,25-diOHC production, was downregulated in tumours despite elevated circulating 7α,25-diOHC. This opposing direction argues against the tumour being the principal source of the circulating metabolite and points instead to extra-tumoural contributions, potentially from the liver, adipose tissue or circulating immune cells.

These findings extend previous work linking oxysterols to colorectal neoplasia. A recent prospective study linked circulating oxysterols to adenoma risk [18], but did not measure the 7-KC/7β-OHC redox couple or 7α,25-diOHC. By analysing this couple as a defined redox system in established carcinoma, our data reveal a layer of oxysterol biology not captured by conventional single-metabolite profiling. The marked shift in the circulating 7-KC/7β-OHC ratio, despite the absence of significant change in 7-KC alone, shows how the internal balance of a linked metabolite pair can reveal a coordinated metabolic state that is less apparent from either component considered individually. The consistent suppression of LXR receptors (*NR1H3*, *NR1H2*) and their target *ABCA1* further suggests attenuation of oxysterol-responsive signalling in established carcinoma.

Several limitations should be considered. First, the plasma cohort is small; although effect sizes were large, consistent across all CRC patients and robust to resampling, the study is not powered to estimate population-level variability or clinical performance reliably, and residual risk of chance or cohort-specific effects cannot be excluded. Accordingly, the circulating findings require independent validation before any biomarker or clinical implication can be considered. Second, cases and controls were not matched for all potential confounders or all pre-analytical variables, as samples originated from different sources, and clinical and metabolic information was incomplete, particularly for the healthy controls. We therefore cannot exclude confounding by age, sex, diet, BMI, fasting status, medication use, including lipid-lowering therapy, diabetes or other metabolic factors, which may contribute to baseline variability. Third, the TCGA and CPTAC analyses were performed in independent cohorts and therefore do not permit direct correlation of tumour *HSD11B2* expression or protein abundance with plasma metabolites within the same individuals. Fourth, because TCGA data derive from bulk tissue, expression differences may partly reflect variation in tumour purity or stromal composition; definitive cell-intrinsic attribution will require cell-resolved and functional analyses. Fifth, although the CPTAC analysis supports HSD11B2 suppression at the protein level, protein abundance does not necessarily establish enzyme activity. Functional validation, including direct measurement and perturbation of HSD11B2 activity, will be required to determine whether altered HSD11B2 activity contributes to the circulating shift in the 7-KC/7β-OHC ratio. Finally, the cross-sectional design precludes assessment of temporal dynamics during disease progression and treatment. Validation in larger, prospectively annotated cohorts, ideally with paired plasma and tumour sampling, will be required to establish whether the observed circulating phenotype is generalisable in CRC and whether it has prognostic, stratification or treatment-response relevance.

## 5. Conclusions

In summary, we identify a selective shift in the plasma 7-oxysterol redox axis in CRC that is not mirrored by tumour-local metabolite changes, but is directionally consistent with tumour-associated transcriptional remodelling of oxysterol metabolism and may reflect integrated contributions from multiple tissues. These findings indicate that the 7-KC/7β-OHC ratio reflects the balance between biochemically linked circulating oxysterols in CRC and highlights defined oxysterol redox systems as a biologically informative layer of cancer metabolism. These findings warrant validation in larger, prospectively matched cohorts.

## Figures and Tables

**Figure 1:**
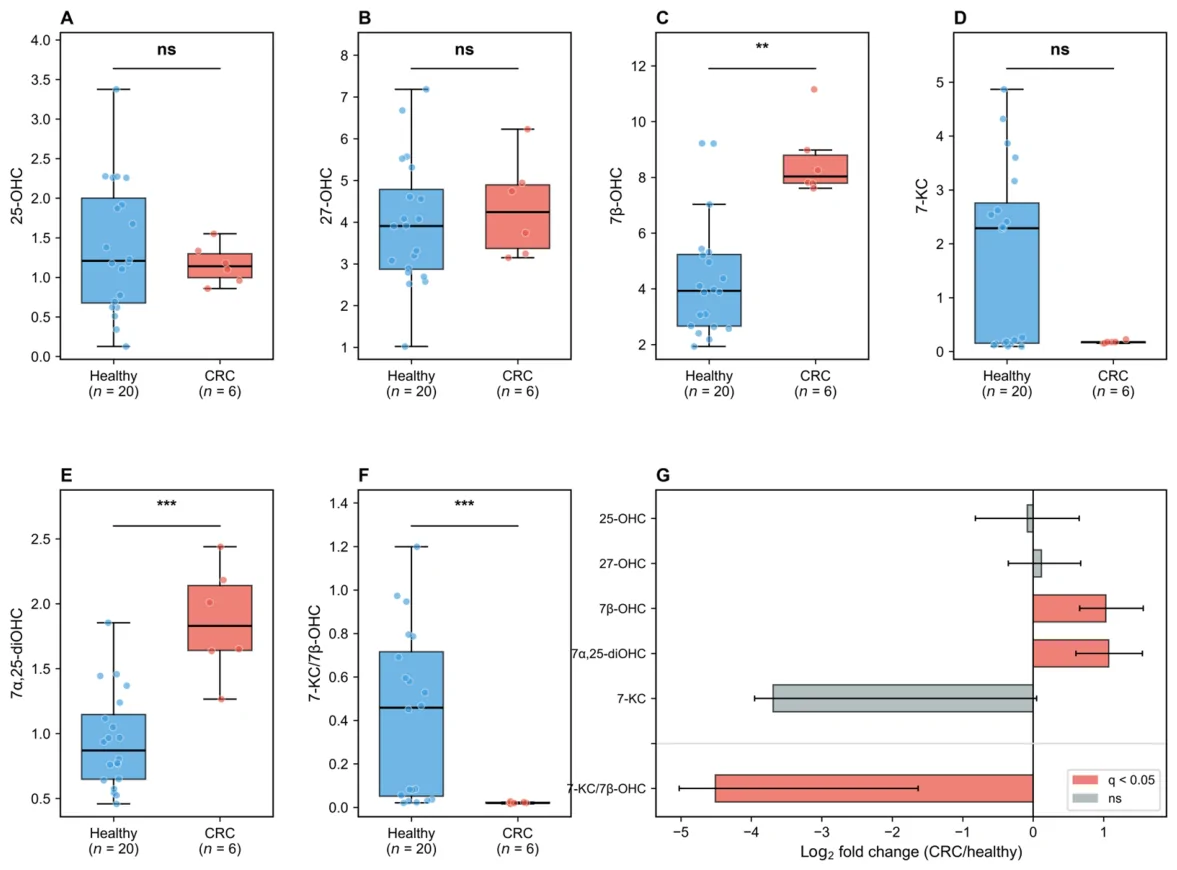
Plasma oxysterol profiles in colorectal cancer. (**A**–**F**) Boxplots of cholesterol-normalised oxysterol concentrations in plasma from healthy controls (*n* = 20) and CRC patients (*n* = 6): (**A**) 25-OHC, (**B**) 27-OHC, (**C**) 7β-OHC, (**D**) 7-KC, (**E**) 7α,25-diOHC, (**F**) 7-KC/7β-OHC ratio. Individual data points are shown. ** q ≤ 0.01, *** q ≤ 0.001; ns, not significant; two-sided Mann–Whitney U tests with Benjamini–Hochberg FDR correction. Significance annotations in panels A-F are based on FDR-adjusted q-values. (**G**) Forest plot of log_2_ fold changes (CRC/healthy controls) with 95% confidence intervals. Red bars indicate FDR-corrected significance (q < 0.05); grey bars indicate non-significance.

**Figure 2:**
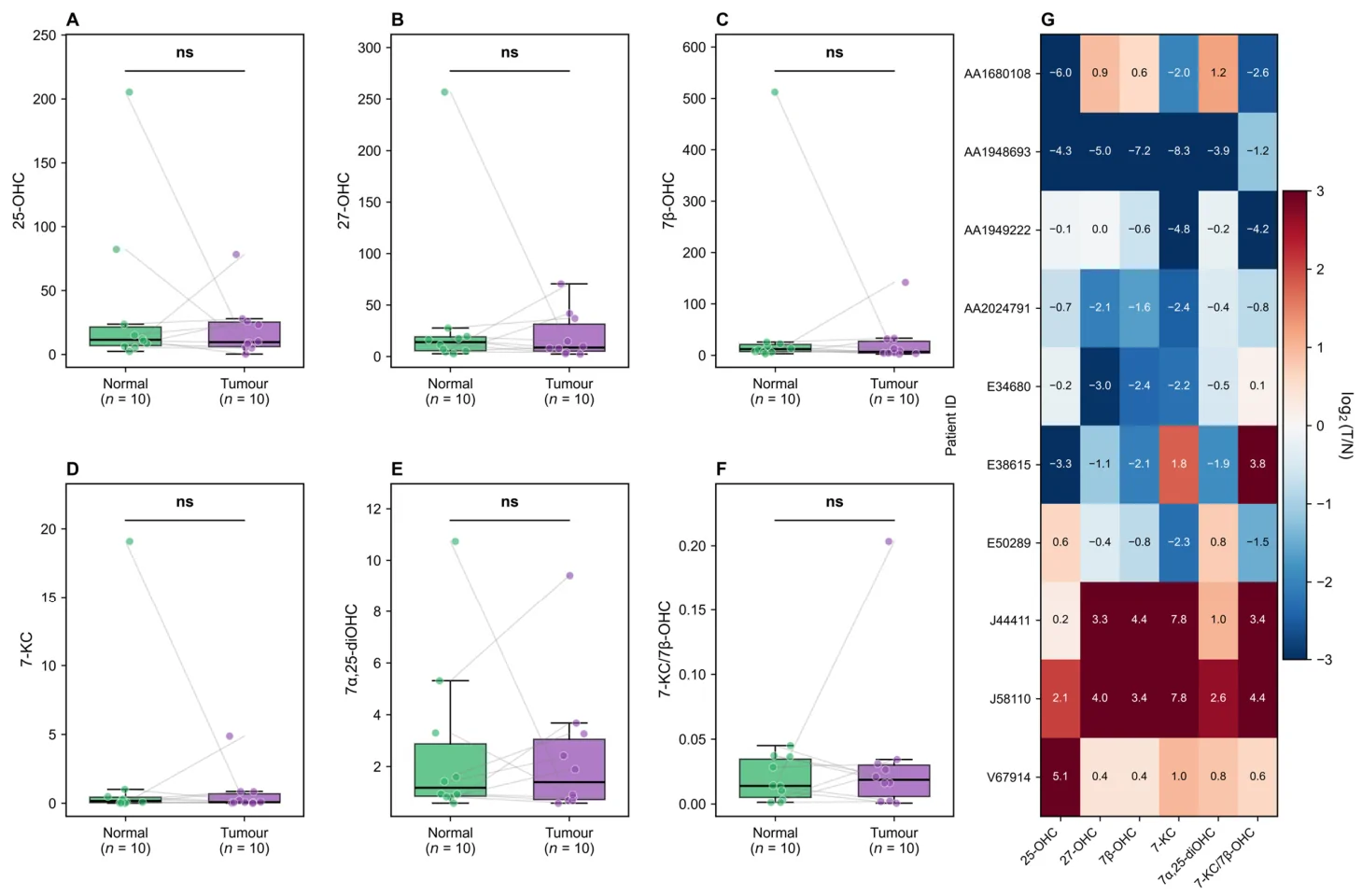
Oxysterol profiles in paired colorectal tumour and adjacent normal tissues. (**A**–**F**) Paired boxplots of cholesterol-normalised oxysterol concentrations in tumour and adjacent normal tissue (*n* = 10 pairs): (**A**) 25-OHC, (**B**) 27-OHC, (**C**) 7β-OHC, (**D**) 7-KC, (**E**) 7α,25-diOHC, (**F**) 7-KC/7β-OHC ratio. Grey lines connect matched samples. Two-sided Wilcoxon signed-rank tests were used; all comparisons were non-significant (ns). (**G**) Per-patient heatmap of log_2_(tumour/normal) fold changes for each oxysterol and the 7-KC/7β-OHC ratio. Exact log_2_ fold-change values are shown within each cell. Colour scale is fixed to ±3 log_2_FC to preserve visual resolution across moderate changes; values outside this range are saturated for colour display only.

**Figure 3:**
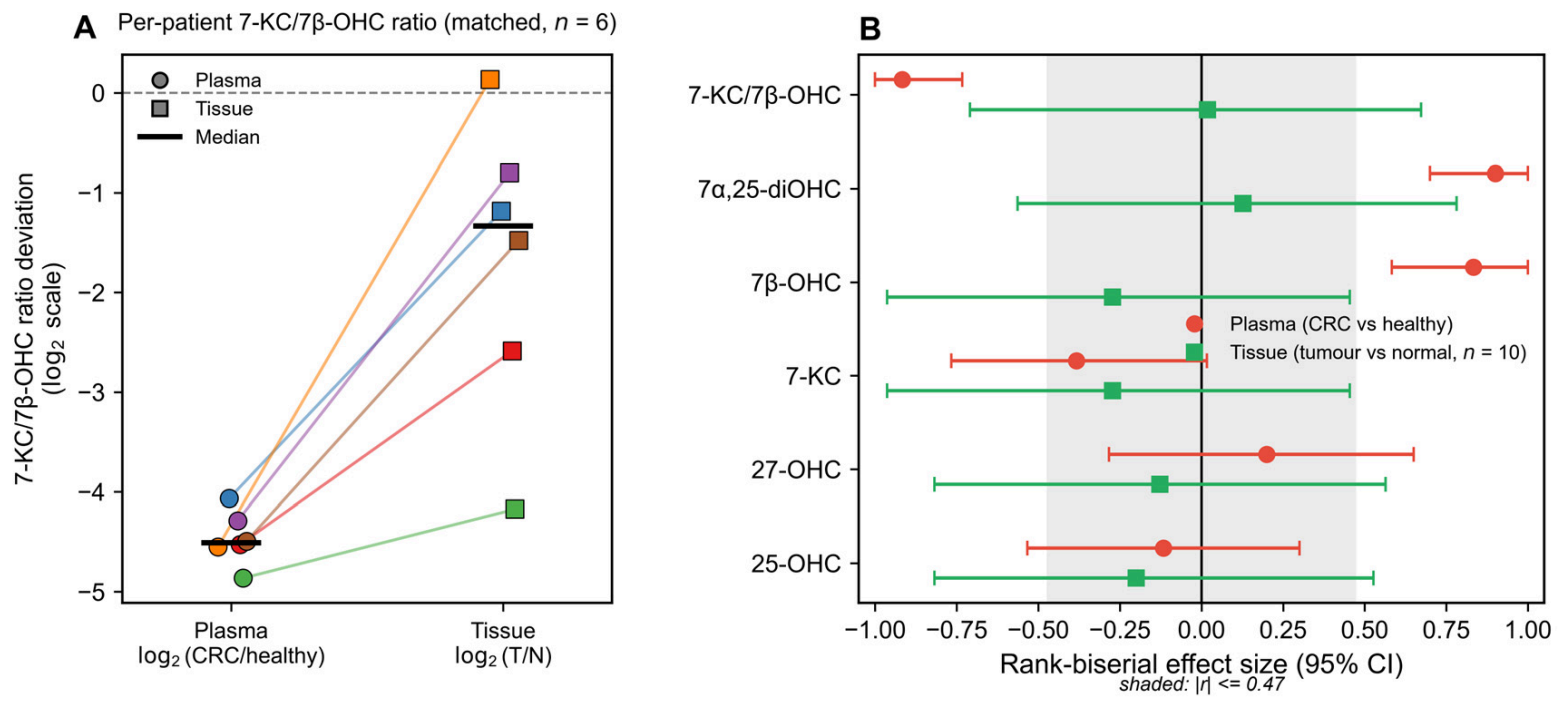
Plasma and tumour-tissue 7-KC/7β-OHC ratio. (**A**) Per-patient slope plot of the 7-KC/7β-OHC ratio in the six CRC patients with matched plasma and tissue. Plasma values are shown as log_2_(patient/healthy control median) and tissue values as log_2_(tumour/adjacent normal). Circles indicate plasma, squares indicate tissue and colours identify individual patients. Horizontal bars indicate group medians. (**B**) Rank-biserial effect sizes (r) with bootstrap 95% confidence intervals, estimated independently for plasma (CRC vs. healthy controls, *n* = 6 vs. *n* = 20; red circles) and tissue (tumour vs. adjacent normal, *n* = 10 pairs; green squares). Grey shading indicates |r| ≤ 0.47. Panel A is restricted to the six patients with both sample types; Panel B uses all ten available tissue pairs for the tissue effect-size estimates.

**Figure 4:**
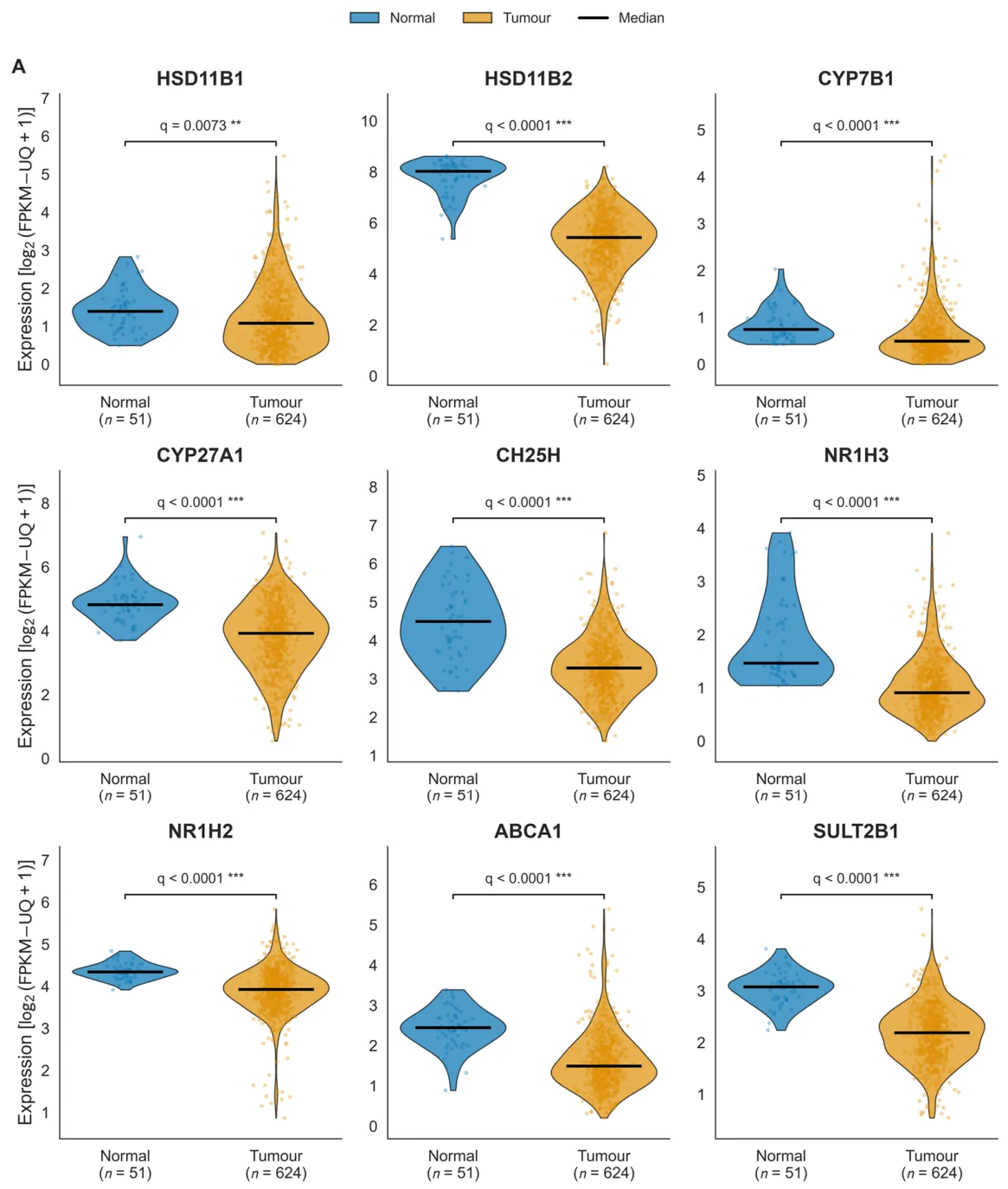

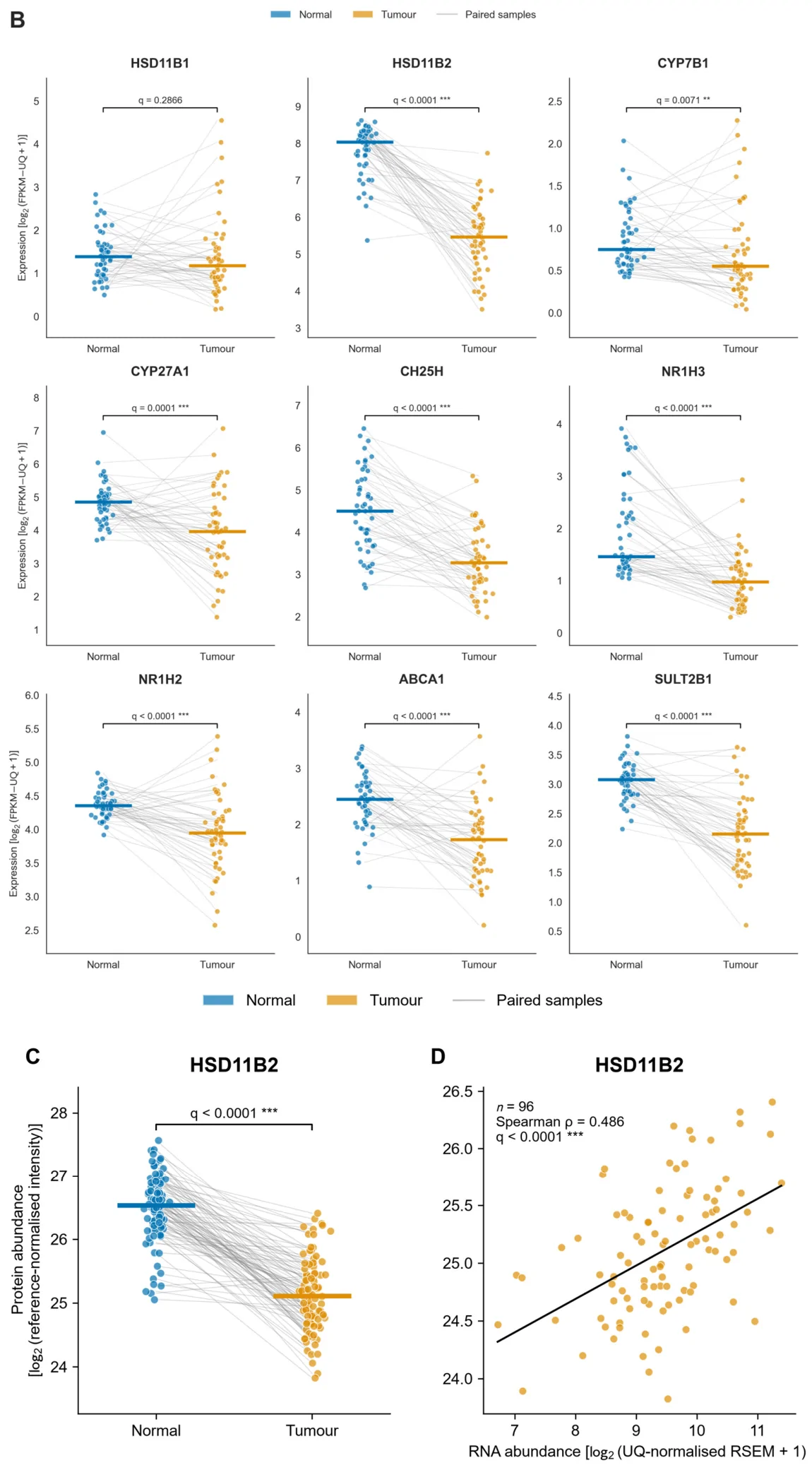
TCGA COAD/READ expression of oxysterol-related genes and independent CPTAC validation of HSD11B2 protein suppression. (**A**) Unpaired tumour versus adjacent normal tissue expression for nine oxysterol-related genes (log_2_(FPKM-UQ + 1); patient-level analysis; normal *n* = 51; tumour *n* = 624). (**B**) Paired tumour-normal analysis (*n* = 50 matched pairs) with lines connecting matched samples. Horizontal bars indicate medians. For (**A**,**B**), q-values are Benjamini–Hochberg FDR-adjusted; two-sided Mann–Whitney U tests were used in (**A**) and two-sided Wilcoxon signed-rank tests in (**B**). TCGA effect sizes and log_2_ fold-changes are reported in Table S2. (**C**) Paired HSD11B2 protein abundance in CPTAC COAD tumour and matched normal-adjacent tissue (*n* = 96 pairs). Lines connect matched samples. (**D**) Association between HSD11B2 transcript and protein abundance in CPTAC COAD tumours with matched measurements (*n* = 96); Spearman rank correlation. For (**C**,**D**), q-values are Benjamini–Hochberg adjusted across the two CPTAC validation analyses. ** q ≤ 0.01; *** q ≤ 0.001.

## Data Availability

The TCGA COAD/READ RNA-seq (FPKM-UQ) and clinical data analysed in this study are publicly available via the UCSC Xena platform (TCGA-COADREAD cohort) [27]. CPTAC COAD proteomic and transcriptomic data are publicly available through the Clinical Proteomic Tumour Analysis Consortium; processed data used in this study were accessed through the cptac Python package [29,30]. The raw oxysterol and cholesterol data and analysis code supporting the conclusions of this article will be made available by the authors on request.

## Supplementary Materials

The following supporting information can be downloaded at https://www.mdpi.com/article/10.3390/metabo16100716/s1, Figure S1: Plasma cholesterol levels; Figure S2: Multiple testing correction for plasma oxysterol comparisons; Figure S3: Sample-level plasma oxysterol profiles; Figure S4: Leave-one-out sensitivity analysis of the plasma 7-KC/7β-OHC ratio; Figure S5: Multivariate analysis of plasma oxysterols; Figure S6: Oxysterol pools and plasma-tissue correlations; Figure S7: TCGA COAD/READ oxysterol-pathway gene expression stratified by MSI status and tumour stage; Figure S8: CPTAC subgroup analyses of *HSD11B2* expression and protein abundance; Table S1: Available demographic and clinical characteristics of the study cohorts; Table S2: Differential expression of oxysterol-related genes in TCGA COAD/READ; Table S3: Direct comparison of oxysterol-related gene expression between TCGA colorectal cancer subgroups.

## Abbreviations

The following abbreviations are used in this manuscript:

7α,25-diOHC	7α,25-dihydroxycholesterol
7-KC	7-ketocholesterol
7β-OHC	7β-hydroxycholesterol
25-OHC	25-hydroxycholesterol
27-OHC	27-hydroxycholesterol
ABCA1	ATP-binding cassette transporter A1
CH25H	cholesterol 25-hydroxylase
CI	confidence interval
COAD	colon adenocarcinoma
CPTAC	Clinical Proteomic Tumour Analysis Consortium
CRC	colorectal cancer
CYP7B1	cytochrome P450 family 7 subfamily B member 1
CYP27A1	cytochrome P450 family 27 subfamily A member 1
EBI2/GPR183	Epstein–Barr virus-induced gene 2/G protein-coupled receptor 183
FDR	false discovery rate
FPKM-UQ	fragments per kilobase of transcript per million mapped reads, upper-quartile normalised
HPLC	high-performance liquid chromatography
HSD11B1	11β-hydroxysteroid dehydrogenase type 1
HSD11B2	11β-hydroxysteroid dehydrogenase type 2
LC-MS/MS	liquid chromatography-tandem mass spectrometry
LLOQ	lower limit of quantification
LOD	limit of detection
log_2_FC	log_2_ fold change
LOO	leave-one-out
LXR	liver X receptor
MSI-H	microsatellite instability-high
MSS	microsatellite stable
NAD^+^/NADH	nicotinamide adenine dinucleotide (oxidised/reduced)
NADPH	nicotinamide adenine dinucleotide phosphate (reduced)
NR1H2	nuclear receptor subfamily 1 group H member 2 (LXRβ)
NR1H3	nuclear receptor subfamily 1 group H member 3 (LXRα)
PC1	first principal component
q	Benjamini–Hochberg false discovery rate-adjusted *p*-value
READ	rectum adenocarcinoma
SULT2B1	sulfotransferase family 2B member 1
TCGA	The Cancer Genome Atlas
UCSC	University of California, Santa Cruz
