# Supplementary material for: A Shift in the Plasma 7-Oxysterol Redox Axis in Colorectal Cancer
Source: Metabolites. 2026 Sep 26;16(10):716. doi: 10.3390/metabo16100716 (PMC13706939; doi:10.3390/metabo16100716)
Supplement: Supplementary file 1 [file metabolites-16-00716-s001.zip › metabolites-4504878-supplementary.pdf]

Supplementary figure legends

Figure S1. Plasma cholesterol levels. Boxplot showing total plasma cholesterol concentrations in healthy controls (*n* = 20) and CRC patients (*n* = 6). Individual data points are shown. Statistical comparison used a two-sided Mann-Whitney U test; *p* = 0.072.

Figure S2. Multiple testing correction for plasma oxysterol comparisons. Raw p-values, Bonferroni-corrected p-values and Benjamini-Hochberg FDR q-values are shown for plasma comparisons between healthy controls (*n* = 20) and CRC patients (*n* = 6). Raw p-values were calculated using two-sided Mann-Whitney U tests. Benjamini-Hochberg FDR $q < 0.05$ was considered significant after correction; raw and Bonferroni-adjusted p-values are shown for transparency.

Figure S3. Sample-level plasma oxysterol profiles. Heatmap showing Z-scored oxysterol concentrations for each individual plasma sample. Rows are ordered by group; healthy controls (*n* = 20) and CRC patients (*n* = 6), separated by a horizontal line. Columns show individual oxysterols and the 7-KC/7β-OHC ratio.

Figure S4. Leave-one-out sensitivity analysis of the plasma 7-KC/7β-OHC ratio. (**A**) Leave-one-out (LOO) sensitivity analysis of the CRC-control difference in the 7-KC/7β-OHC ratio. Each healthy control (*n* = 20; blue) and each CRC patient (*n* = 6; red) was sequentially excluded, and the CRC-control comparison was re-tested using a two-sided Mann-Whitney U test. Points show $-\log_{10}(p)$ values; the dashed line indicates $p = 0.05$ and the dotted line separates healthy-LOO from CRC-LOO iterations. (**B**) Distribution of the $\log_2$-transformed plasma ratio across healthy controls and CRC patients. Healthy controls are displayed using a descriptive median split to visualise within-group heterogeneity (lower half in blue, upper half in orange). The split is descriptive only and was not used for statistical testing.

Figure S5. Multivariate analysis of plasma oxysterols. (**A**) Principal component analysis (PCA) of plasma oxysterol profiles from healthy controls (*n* = 20; blue circles) and CRC patients (*n* = 6; red squares), performed using the five directly measured oxysterols (25-OHC, 27-OHC, 7β-OHC, 7-KC and 7α,25-diOHC). The derived 7-KC/7β-OHC ratio was not included in the PCA. PC1 and PC2 explained 44.4% and 32.2% of the variance, respectively. (**B**) Loadings of the five measured oxysterols on PC1 and PC2. (**C**) Spearman correlation matrix of plasma oxysterols and the 7-KC/7β-OHC ratio. Spearman correlation coefficients are shown within each cell.

Figure S6. Oxysterol pools and plasma-tissue correlations. (**A**) Bar plot comparing oxo (7-KC), hydroxylated (7β-OHC + 7α,25-diOHC) and total measured 7-oxysterol pools (7-KC + 7β-OHC + 7α,25-diOHC) in plasma from healthy controls (blue, *n* = 20) and CRC patients (red, *n* = 6). Bars show group medians of cholesterol-normalised concentrations. Statistical comparisons used two-sided Mann-Whitney U tests; significance labels indicate raw p-values. The hydroxylated pool is significantly elevated in CRC (***$p < 0.001$), while the oxo pool shows no significant change (ns),

resulting in increased total 7-oxysterols (*p < 0.05). (**B**) Spearman correlation coefficients between plasma and tissue oxysterol concentrations in matched CRC cases (n = 6). Bars show Spearman rho values; colours indicate whether raw p < 0.05.

Figure S7. TCGA COAD/READ oxysterol-pathway gene expression stratified by MSI status and tumour stage. (**A**) Tumour-versus-normal expression differences for nine oxysterol-related genes. Overall values compare all tumours with the full normal set; MSI strata compare MSS or MSI-H tumours against the full normal set. Stage strata compare early-stage (I-II) or late-stage (III-IV) tumours against the full normal set. Values indicate differences in mean $\log_2$(FPKM-UQ + 1) expression between tumour and normal groups. **(B)** Direct tumour-subgroup comparisons for the same nine genes. Values indicate the difference in mean $\log_2$(FPKM-UQ + 1) expression for MSI-H minus MSS tumours and late-stage minus early-stage tumours. Negative values therefore indicate lower expression in MSI-H or late-stage tumours, respectively. Two-sided Mann-Whitney U tests were used for unpaired comparisons. Benjamini-Hochberg correction was applied across the nine-gene panel separately for each comparison. Significance is indicated as *q < 0.05, **q < 0.01 and ***q < 0.001.

Figure S8. CPTAC subgroup analyses of *HSD11B2* expression and protein abundance. **(A)** *HSD11B2* transcript abundance in early-stage (I-II; n = 54) and late-stage (III-IV; n = 51) CPTAC COAD tumours. **(B)** HSD11B2 protein abundance in early-stage (n = 49) and late-stage (n = 48) tumours. **(C)** Patient-level paired tumour-normal HSD11B2 protein difference stratified by microsatellite instability status (MSS, n = 74; MSI-H, n = 21). More negative values indicate greater tumour-associated reduction in HSD11B2 protein abundance. Points represent individual patients and horizontal bars indicate medians. Two-sided Mann-Whitney U tests were used for the three subgroup comparisons, with Benjamini-Hochberg correction applied across the three analyses shown in panels A-C.

Table S1. Available demographic and clinical characteristics of the study cohorts. **(A)** Individual demographic and available clinical characteristics of the 10 CRC patients contributing paired tumour and adjacent-normal tissue samples. Available information includes age, sex, BMI, smoking history, diabetes status, fasting status, recorded current medication, past medical history, tumour location and pathological stage. Plasma availability and inclusion in the plasma analysis are indicated; plasma from patient J58110 was excluded because of clinically diagnosed liver cirrhosis. **(B)** Age and sex of the 20 healthy plasma donors. Clinical and metabolic information beyond age and sex was not available for the healthy control cohort. Within the CRC cohort, the completeness of clinical information varied across variables and participants.

Table S2. Differential expression of oxysterol-related genes in TCGA COAD/READ. Summary statistics for nine genes involved in oxysterol metabolism. Normal n and tumour n indicate unique patient counts for unpaired analysis after averaging multiple aliquots per patient within tissue type. Log_2FC_mean represents the difference in mean $\log_2$(FPKM-UQ + 1) expression between tumour and normal groups.

Log_2FC_median represents the difference in median log_2(FPKM-UQ + 1) expression between tumour and normal groups as a robustness check. Unpaired q and paired q indicate Benjamini-Hochberg FDR-adjusted p-values from unpaired and paired analyses, respectively. Cliff's δ indicates the non-parametric effect size, ranging from -1 to +1. Effect categories are based on predefined thresholds (small: $|\delta| < 0.33$; medium: 0.33-0.47; large: > 0.47).

Table S3. Direct comparison of oxysterol-related gene expression between TCGA colorectal cancer subgroups. Patient-level tumour expression of nine oxysterol-related genes was compared directly between MSI-H and MSS tumours and between late-stage (III-IV) and early-stage (I-II) tumours. Expression values are log_2(FPKM-UQ + 1); where multiple tumour aliquots were available for a patient, expression values were averaged before analysis. Mean and median differences are defined as comparison group minus reference group (MSI-H minus MSS and late minus early). Cliff's δ is reported as an effect-size measure, with negative values indicating lower expression in the comparison group. P values were calculated using two-sided Mann-Whitney U tests. Benjamini-Hochberg correction was applied separately across the nine-gene panel for each clinical comparison.

Supplementary Figure S1

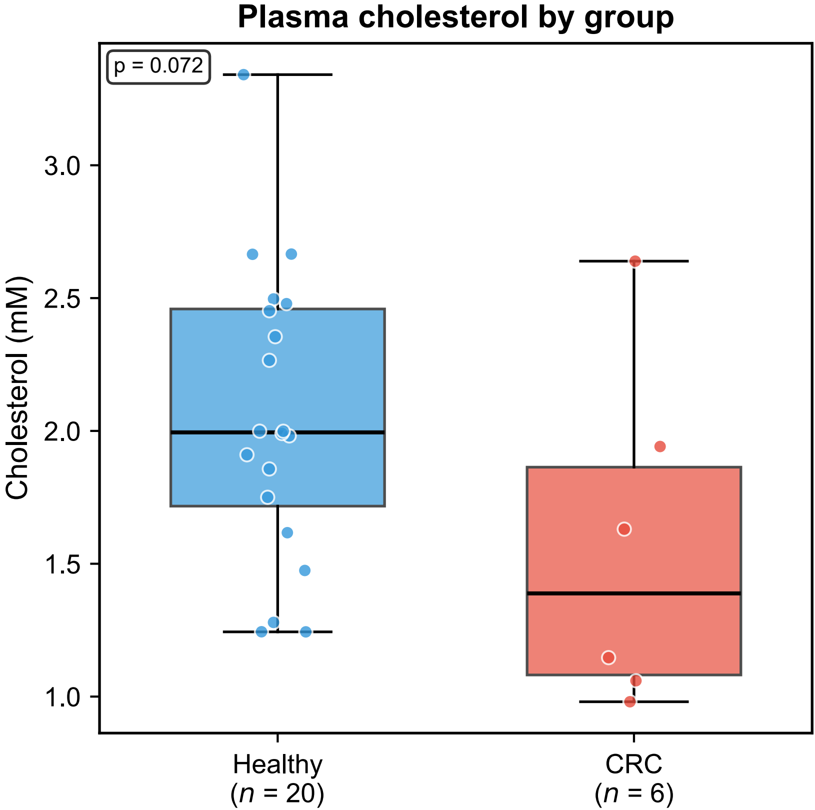

Supplementary Figure S2

Multiple testing correction

Marker	Raw p	Bonferroni	Sig?	BH FDR	Sig?
25-OHC	0.6999	1.000	No	0.700	No
27-OHC	0.4942	1.000	No	0.593	No
7β-OHC	0.0011	0.007	Yes	0.002	Yes
7-KC	0.1756	1.000	No	0.263	No
7α,25-diOHC	0.0003	0.002	Yes	0.001	Yes
7-KC/7β-OHC	0.0002	0.001	Yes	0.001	Yes

Supplementary Figure S3

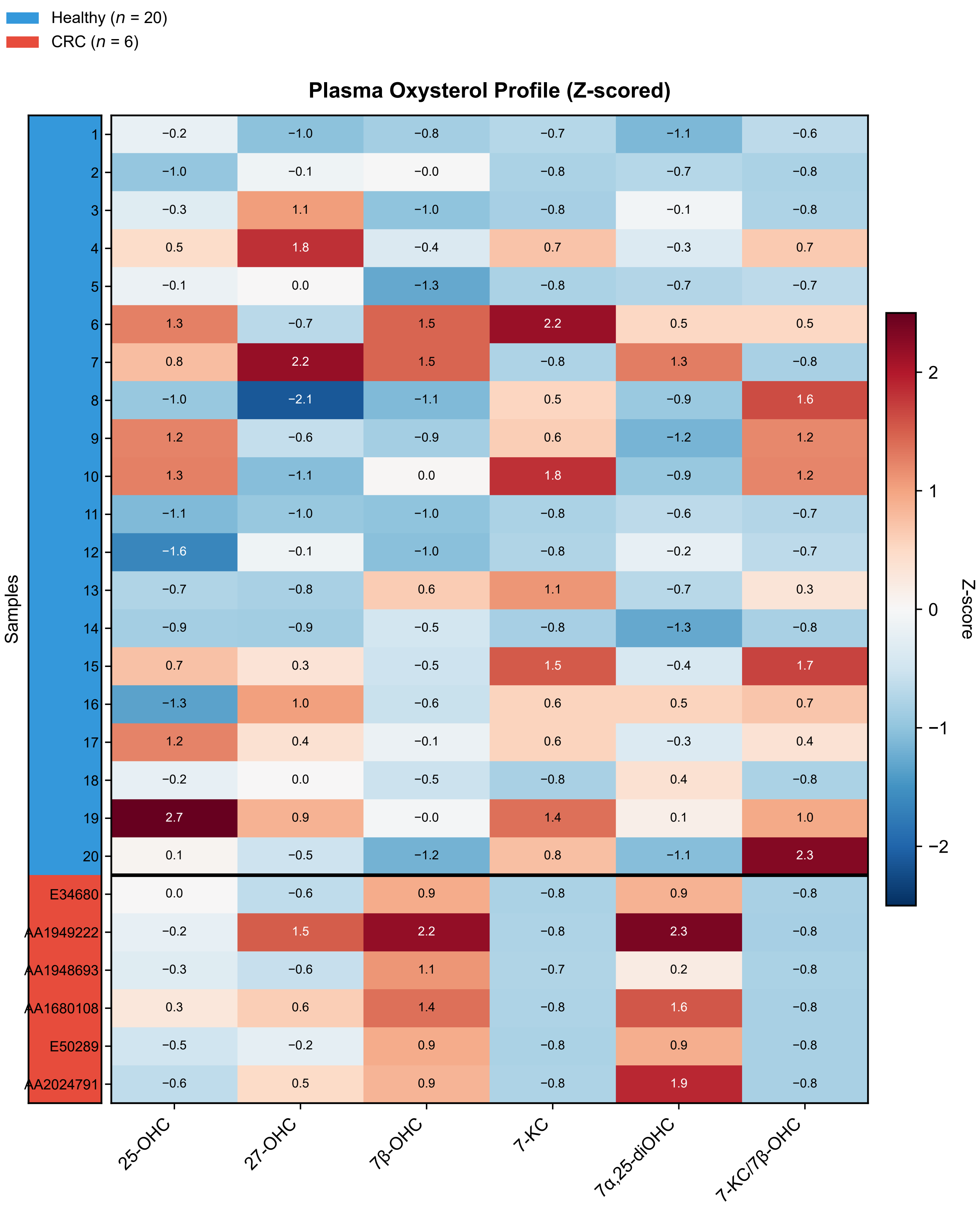

Supplementary Figure S4

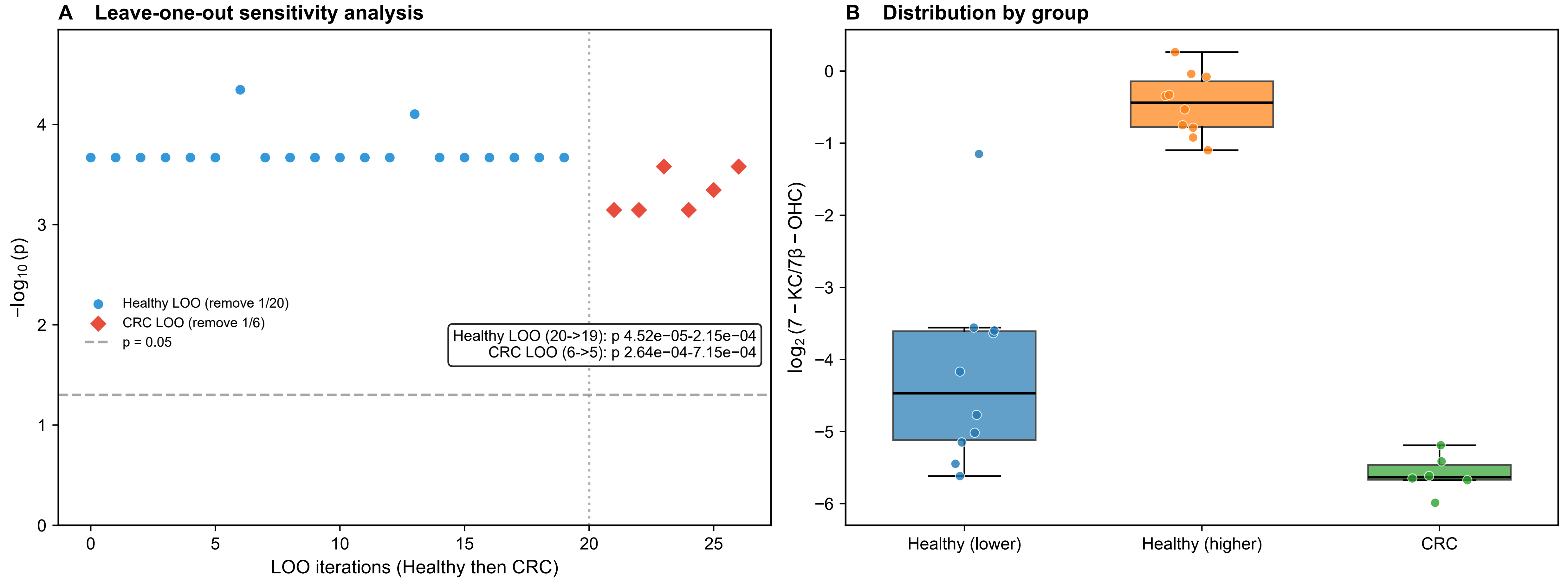

Supplementary Figure S5

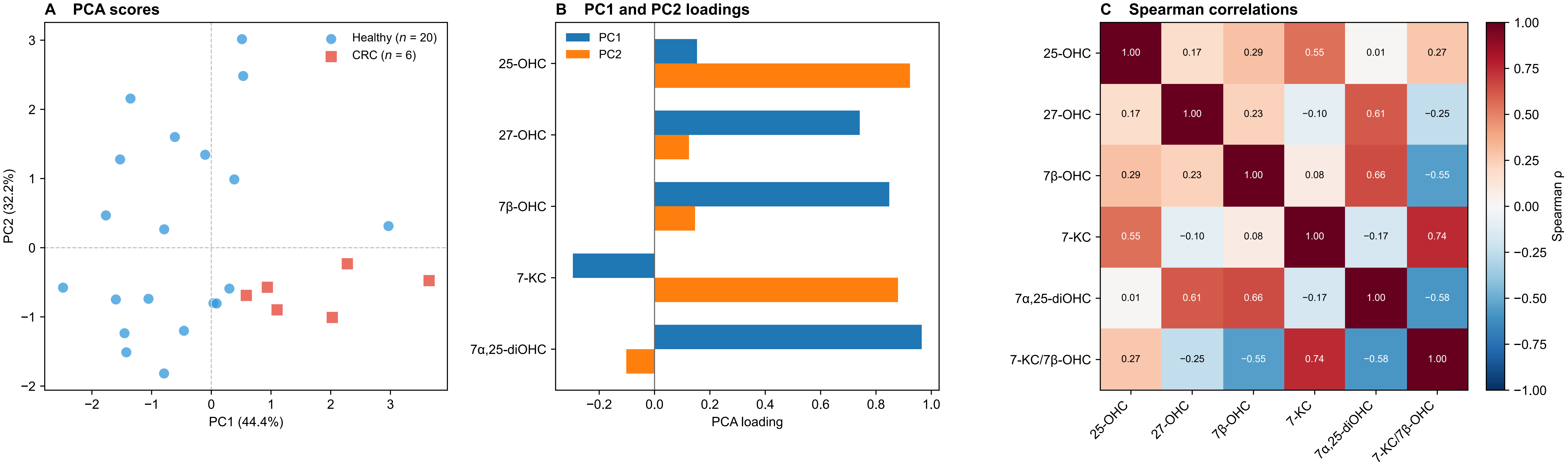

	25-OHC	27-OHC	7β-OHC	7-KC	7α,25-diOHC	7-KC/7β-OHC
25-OHC	1.00	0.17	0.29	0.55	0.01	0.27
27-OHC	0.17	1.00	0.23	−0.10	0.61	−0.25
7β-OHC	0.29	0.23	1.00	0.08	0.66	−0.55
7-KC	0.55	−0.10	0.08	1.00	−0.17	0.74
7α,25-diOHC	0.01	0.61	0.66	−0.17	1.00	−0.58
7-KC/7β-OHC	0.27	−0.25	−0.55	0.74	−0.58	1.00

Supplementary Figure S6

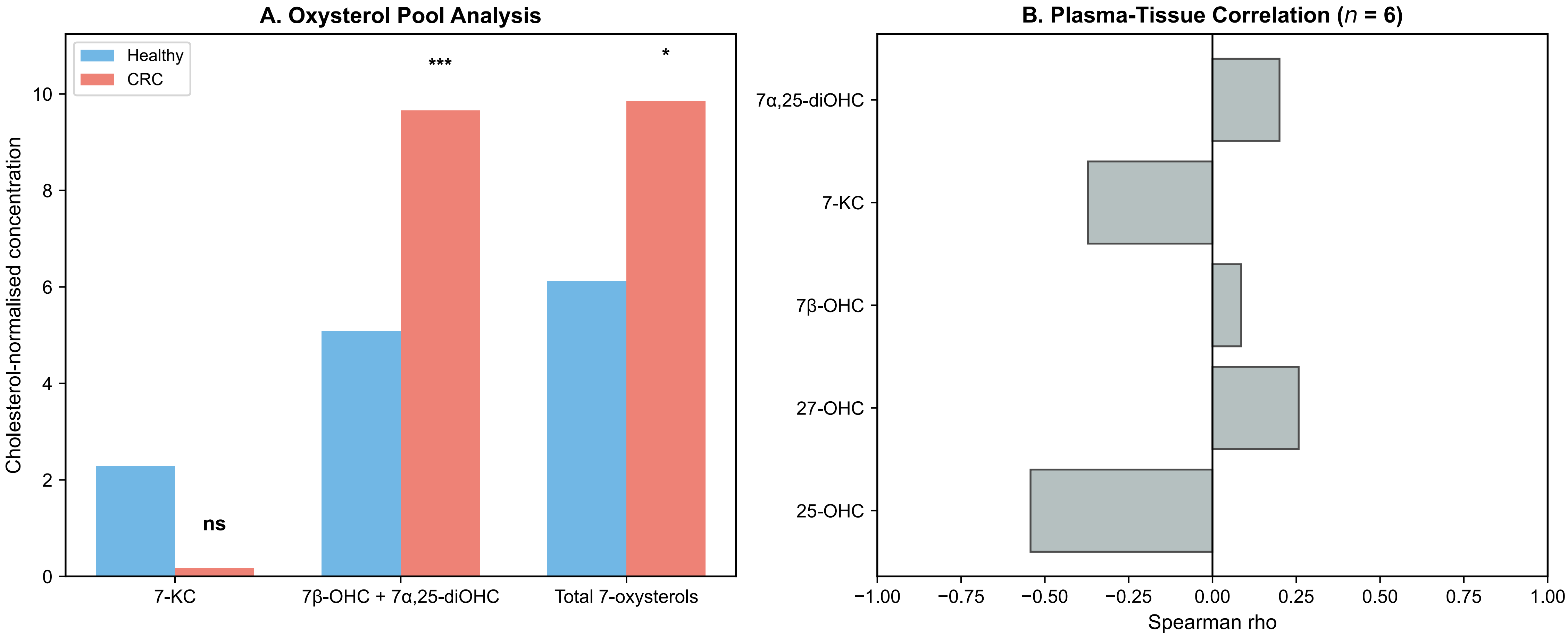

Supplementary Figure S7

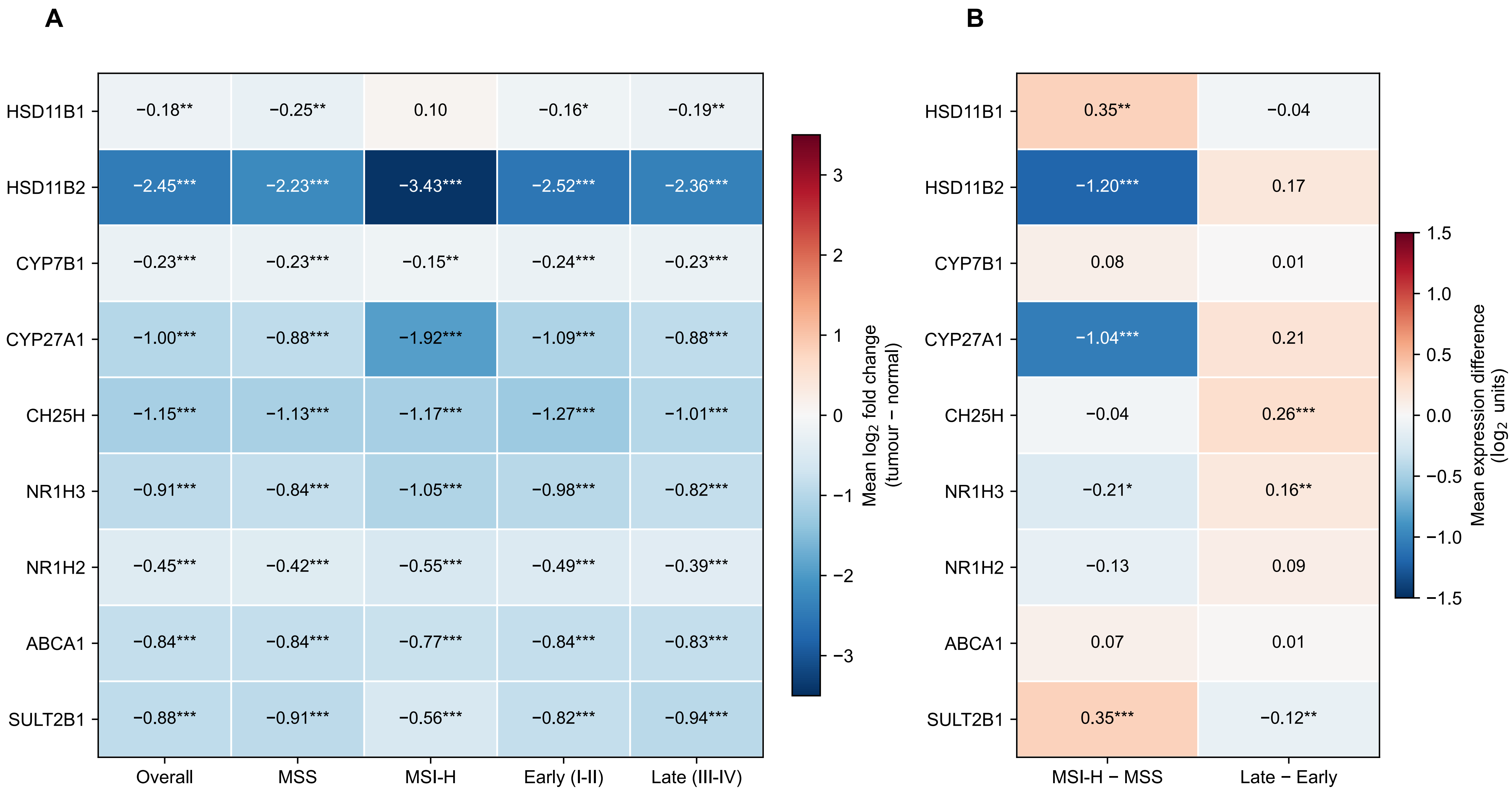

Supplementary Figure S8

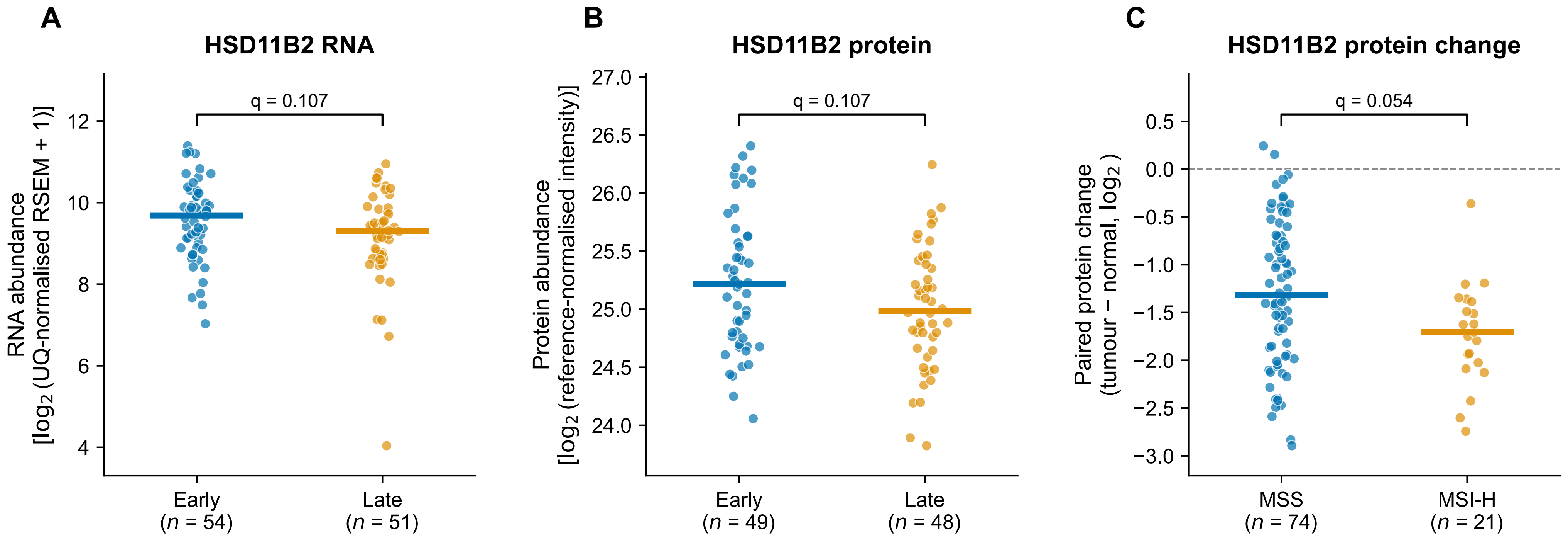

Supplementary Table S1

A. Colorectal cancer cohort

Patient ID	Age (years)	Sex	BMI (kg/m²)	Smoking history	Fasting status	Diabetes status	Recorded current medication	Recorded past medical history	Tumour location	Recorded pathological staging	AJCC stage group	Plasma status
E34680	87	M	25.9	Not available	Yes (6 h)	Not diabetic	Nitrofurantoin; Finasteride; Ramipril; Levothyroxine; Tamsulosin; Senokot; Lactulose	Hypertension	Sigmoid colon	pT2 pN0 pM0	I	Included
AA1949222	71	M	30.9	Never smoked	Yes (6 h)	Not diabetic	Enoxaparin	Cardiomegaly; ischaemic heart disease	Rectum	pT2 pN0 R0	I	Included
AA1948693	61	M	25.9	Never smoked	Yes (6 h)	Not diabetic	None recorded	None recorded	Lower rectum	pT3 pN0 R0	II	Included
AA1680108	36	F	23.5	Never smoked	Yes (6 h)	Not diabetic	None recorded	None recorded	Sigmoid colon	pT3 pN1b R0	III	Included
E50289	78	M	22.6	Not available	Not available	Not diabetic	Not available	Monoclonal gammopathy; prostate cancer	Right colon	pT4a/b pN1b R0	III	Included
AA2024791	42	F	20.1	Never smoked	Not available	Not diabetic	Not available	None recorded	Transverse colon	pT4a pN0 pM1c (perforated)	IV	Included
J58110	63	M	18.9	Not available	Not available	Type 1 diabetes	Not available	Liver cirrhosis; Type 1 diabetes	Caecum	pT3 pN0 R0	II	Available; excluded from plasma analysis
J44411	76	F	31.1	Former smoker; ceased >12 months before surgery	Not available	Not diabetic	Doxazosin; Losartan; Atorvastatin; Bisoprolol	Hypotension; pacemaker in situ	Mid ascending colon	pT3 pN2b	III	Not available
E38615	70	F	37.3	Never smoked	Yes (6 h)	Not diabetic	Omeprazole	None recorded	Ascending colon and low rectum	pT3 pN0 R0	II	Not available
V67914	74	M	Not available	Non-smoker	Yes (6 h)	Not diabetic	Lisinopril; Amlodipine; Simvastatin	Hypertension	Distal transverse colon	pT1 pN0	I	Not available

B. Healthy plasma controls

Healthy donor	Sex	Age
1	F	70
2	F	73
3	F	77
4	F	71
5	M	69
6	F	71
7	F	71
8	M	78
9	M	77
10	F	71
11	F	75
12	M	76
13	M	72
14	M	72
15	F	76
16	M	73
17	F	79
18	F	73
19	M	76
20	M	75

Supplementary Table S2

Gene	Function	Normal (n)	Tumour (n)	Paired (n)	log_2FC_mean	log_2FC_median	Unpaired q	Paired q	Cliff's δ	Effect
HSD11B2	7β-OHC to 7-KC	51	624	50	-2.45	-2.56	< 0.0001	< 0.0001	-0.94	large
HSD11B1	7-KC to 7β-OHC	51	624	50	-0.18	-0.32	0.0073	0.2866	-0.23	small
CYP7B1	7α,25-diOHC synthesis	51	624	50	-0.23	-0.26	< 0.0001	0.007116	-0.43	medium
CYP27A1	27-OHC synthesis	51	624	50	-1.00	-0.89	< 0.0001	0.0001	-0.58	large
CH25H	25-OHC synthesis	51	624	50	-1.15	-1.22	< 0.0001	< 0.0001	-0.64	large
NR1H3	LXRα (oxysterol receptor)	51	624	50	-0.91	-0.56	< 0.0001	< 0.0001	-0.70	large
NR1H2	LXRβ (oxysterol receptor)	51	624	50	-0.45	-0.41	< 0.0001	< 0.0001	-0.65	large
ABCA1	Cholesterol/oxysterol efflux	51	624	50	-0.84	-0.98	< 0.0001	< 0.0001	-0.71	large
SULT2B1	Oxysterol sulfation	51	624	50	-0.88	-0.90	< 0.0001	< 0.0001	-0.84	large

Supplementary Table S3

Comparison	Gene	Reference group	Comparison group	Reference n	Comparison n	Reference mean	Comparison mean	Mean difference (log2)	Reference median	Comparison median	Median difference (log2)	Cliff's delta	Effect magnitude	Mann-Whitney P	BH-adjusted q
MSI-H vs MSS	HSD11B1	MSS	MSI-H	386	63	1.178	1.528	0.350	0.985	1.292	0.307	0.225	small	4.239E-03	9.537E-03
MSI-H vs MSS	HSD11B2	MSS	MSI-H	386	63	5.571	4.375	-1.195	5.639	4.381	-1.258	-0.572	large	3.378E-13	3.040E-12
MSI-H vs MSS	CYP7B1	MSS	MSI-H	386	63	0.620	0.704	0.084	0.503	0.584	0.081	0.088	negligible	2.634E-01	3.387E-01
MSI-H vs MSS	CYP27A1	MSS	MSI-H	386	63	3.955	2.919	-1.036	4.013	2.929	-1.084	-0.523	large	2.806E-11	1.263E-10
MSI-H vs MSS	CH25H	MSS	MSI-H	386	63	3.354	3.311	-0.043	3.313	3.166	-0.147	-0.072	negligible	3.587E-01	4.035E-01
MSI-H vs MSS	NR1H3	MSS	MSI-H	386	63	1.089	0.883	-0.206	0.983	0.790	-0.193	-0.213	small	6.600E-03	1.188E-02
MSI-H vs MSS	NR1H2	MSS	MSI-H	386	63	3.940	3.811	-0.129	3.930	3.850	-0.080	-0.130	negligible	9.706E-02	1.456E-01
MSI-H vs MSS	ABCA1	MSS	MSI-H	386	63	1.574	1.647	0.072	1.532	1.659	0.127	0.055	negligible	4.826E-01	4.826E-01
MSI-H vs MSS	SULT2B1	MSS	MSI-H	386	63	2.132	2.483	0.351	2.142	2.482	0.340	0.374	medium	1.967E-06	5.900E-06
Late vs Early	HSD11B1	Early (I-II)	Late (III-IV)	333	268	1.270	1.233	-0.037	1.112	1.072	-0.040	-0.047	negligible	3.232E-01	4.155E-01
Late vs Early	HSD11B2	Early (I-II)	Late (III-IV)	333	268	5.278	5.444	0.166	5.462	5.504	0.042	0.070	negligible	1.395E-01	2.093E-01
Late vs Early	CYP7B1	Early (I-II)	Late (III-IV)	333	268	0.609	0.618	0.009	0.484	0.490	0.006	0.008	negligible	8.617E-01	9.695E-01
Late vs Early	CYP27A1	Early (I-II)	Late (III-IV)	333	268	3.752	3.958	0.207	3.847	4.011	0.164	0.100	negligible	3.564E-02	6.506E-02
Late vs Early	CH25H	Early (I-II)	Late (III-IV)	333	268	3.214	3.470	0.256	3.142	3.419	0.276	0.183	small	1.099E-04	9.892E-04
Late vs Early	NR1H3	Early (I-II)	Late (III-IV)	333	268	0.951	1.113	0.162	0.867	1.025	0.158	0.151	small	1.419E-03	6.384E-03
Late vs Early	NR1H2	Early (I-II)	Late (III-IV)	333	268	3.877	3.971	0.095	3.900	3.970	0.070	0.099	negligible	3.614E-02	6.506E-02
Late vs Early	ABCA1	Early (I-II)	Late (III-IV)	333	268	1.578	1.592	0.014	1.468	1.471	0.003	0.002	negligible	9.747E-01	9.747E-01
Late vs Early	SULT2B1	Early (I-II)	Late (III-IV)	333	268	2.222	2.106	-0.116	2.280	2.103	-0.177	-0.144	negligible	2.371E-03	7.113E-03
